# COVID-19 in jails and prisons: A neglected infection in a marginalized population

**DOI:** 10.1371/journal.pntd.0008409

**Published:** 2020-06-22

**Authors:** Carlos Franco-Paredes, Katherine Jankousky, Jonathan Schultz, Jessica Bernfeld, Kimberly Cullen, Nicolas G. Quan, Shelley Kon, Peter Hotez, Andrés F. Henao-Martínez, Martin Krsak

**Affiliations:** 1 Department of Medicine, Division of Infectious Diseases, University of Colorado, Anschutz Medical Center, Aurora, Colorado, United States of America; 2 Instituto Nacional de Salud, Hospital Infantil de México, Federico Gomez, México City, México; 3 Criminal Defense Attorney, Denver, Colorado, United States of America; 4 UCHealth, Family Medicine, Denver, Colorado, United States of America; 5 Texas Children’s Center for Vaccine Development, Departments of Pediatrics and Molecular Virology & Microbiology, National School of Tropical Medicine, Baylor College of Medicine, Houston, Texas, United States of America; National Institutes of Health, UNITED STATES

The world’s prison population is estimated at around 11 million with rates of incarceration ranging from 698 per 100,000 population in the United States to as low as 16 per 100,000 in the Central African Republic [1]. In the US, there are approximately 2.2 million people incarcerated, representing an estimated 24% of the world’s proportion of incarcerated individuals [2–3]. Estimates as of mid-May 2020 in the US demonstrate that state jails hold 1,230,000 individuals, 625,000 are detained in local jails, and 225,000 in federal jails and prisons [4]. Mass incarceration policies have important collateral damage to prisoners, their families and communities [5]. Investing in social capital, community-building practices, public safety strategies, and violence prevention initiatives represent a more cost-effective approach [4–5]. In communities with steady economic and social breakdown, many groups including African Americans, Hispanic Americans, and white working class without a bachelor’s degree are caught in this web of compound and expanding disadvantage [5–6]. Worldwide, in most settings, poor urban communities experience the highest rates of both incarceration and recidivism [5].

We are enduring a pandemic due to the Severe Acute Respiratory Syndrome Coronavirus 2 (SARS-CoV-2), the novel pathogen that causes the coronavirus disease 2019 (COVID-19) [7]. This zoonotic agent emerged in December 2019 in Wuhan, Hubei, China, and it has already spread to 187 countries causing almost 6 million confirmed cases and more than 360,000 deaths. In the US alone, as of May 29, there have been 1,750,000 confirmed cases with almost 102,201 deaths [7]. All US states have confirmed cases and most have reported deaths. As the pandemic continues to spread across the country, many outbreaks continue to strike high-population density centers, including nursing homes, residential homes, immigration detention centers, and jails and prisons [8].

Like other viral pathogens, SARS-CoV-2 is closely dependent on the complexity of human behavior and human interactions. There are many documented outbreaks of respiratory pathogens in jails and prisons in many countries [9]. Custodial institutions have been the epicenter of outbreaks of infections amongst prisoners amplifying infections at rates far exceeding those in nonincarcerated communities. Highly transmissible viral infections such as measles, mumps, and the novel coronavirus disseminates rapidly among inmates and staff and potentially into the larger community [9]. Overcrowding, insufficient sanitation, poor ventilation, and inadequate healthcare in prisons contribute to enabling these institutions as breeding grounds of infectious disease outbreaks [9–10]. Detention and incarceration of any kind involves large groups of people living in cohorts in confined spaces creating many challenges for curbing the spread of COVID-19 [10]. The number of single rooms in jails or prisons are insufficient to adhere to the recommended isolation and quarantine guidelines and limits the ability to implement strict infection prevention protocols.

The SARS-CoV-2 is able to survive for prolonged periods on materials that are highly prevalent in custodial settings including nonporous surfaces and metallic surfaces complicating disinfection practices. It is exceedingly difficult to comply with established infection prevention protocols recommending repeated disinfection and decontamination of all surfaces in jails and prisons, resulting from the large number of inmates and complex human patterns of interactions between inmates and with the staff [9]. A recent natural experiment inside a cruise ship demonstrated the rapid spread of the SARS-CoV-2 among large crowds inside a closed environment [11]. A total of 696 cumulative cases of COVID-19 and 9 deaths occurred among the 3711 passengers inside the Princess Diamond Cruise berthed at the port of Yokohama, Japan from February 3 to March 1, 2020 [11]. The most recent estimates demonstrate that the basic reproduction number of the SARS-CoV-2 is higher than previously estimated and higher than the influenza virus A/H_1_N_1_ that caused the 1918 to 1919 pandemic [8]. In contrast to influenza viruses, transmission of this coronavirus occurs by those with undetected infection having mild symptoms or asymptomatic infection in up to 20% of cases [12]. Thus, transmission of this viral pathogen in closed spaces with a large presence of individuals increases the frequency of exposure and infection. Without complete social distancing in imprisonment settings, our ability to reduce the transmission dynamics to achieve an R_0_ less than 1 is limited.

Globally, there are widespread concerns about large COVID-19 epidemics sweeping through the incarcerated populations in China, Brazil, India, Indonesia, and several African nations, leading to calls for parole or early release [13]. In the US, there are already thousands of confirmed cases of COVID-19 tied to prisons and jails with many deaths among prisoners and staff (Table 1). These include some of America’s largest outbreaks in the most populated jails including Cook County Jail, Los Angeles County Jail System, Sterling Prison in Colorado, and many others. However, the true extent of the epidemic inside the walls of prisons and jails in the US is largely unknown because of undertesting and underreporting. As the number of cases of COVID-19 continues to spread in the US, it is likely that there will be an increasing number of clusters and outbreaks in carceral settings with implications to the larger community and to the healthcare system [14]. At this point in the pandemic, the capacity to handle a large influx of critically ill patients coming from jails and prisons is limited. Efforts at the federal, state, and local levels are reducing the number of incarcerated individuals [15]. However, without widespread availability of testing in jails and prisons to guide isolation and quarantine practices, inadequate supply of personal protective equipment for inmates including masks, and the revolving door of jails, hampers the ability to block transmission as the current outbreak inside many correctional facilities has uncovered [15].

**Table 1:** Comparison of four different initiatives compiling data on COVID-19 confirmed cases and deaths among prisoners and staff in correctional facilities across the US (Data to May 29, 2020).

Data source	COVID-19 cases among jail-prison residents	COVID-19 cases among staff	COVID-19 deaths among residents	COVID-19 deaths among staff in jails/prisons
UCLA Law COVID-19 Behind Bars^a^	38,616	10,182	470	42
COVID Prison Data^b^	29,519	7402	392	20
CDC Data (May 6 Updated Guidance Correctional Facilities)^c^	4,893	2,778	88	15
The Marshall Project^d^	29,251	7,435	415	33

^a^UCLA Law COVID-19 behind bars data project by Professor Sharon Dolovich, Director–Available from: https://docs.google.com/spreadsheets/d/1X6uJkXXS-O6eePLxw2e4JeRtM41uPZ2eRcOA_HkPVTk/edit#gid=1197647409. [cited 2020 May 29].

^b^COVID Prison Data (Prison Project). Available from: https://covidprisondata.com/. [cited 2020 May 29].

^c^COVID-19 in Correctional and Detention Facilities—United States, February–April 2020 Available from: https://www.cdc.gov/mmwr/volumes/69/wr/mm6919e1.htm. [cited 2020 May 29].

^d^The Marshall Project. Available from: https://www.themarshallproject.org/2020/05/01/a-state-by-state-look-at-coronavirus-in-prisons. [cited 2020 May 29].

In the US, from a societal perspective, the increasingly identified impact of the COVID-19 pandemic among Hispanic Americans and African Americans reveals the prevailing structural violence across localities in the US [15]. Among these communities, the pervasive disparities that result from policies, economic systems, and institutions that limit peoples’ education, access to resources, power relations, and social networks shapes people’s practices and behaviors [15]. These same forces are responsible for maintaining systematic social inequities that result in poor health. The luxury of social distancing is hard to meet for daily workers including those in the service industry. Equally, except for punitive solitary confinement, social distancing is the antithesis of incarceration. Until we institute structural interventions that address the social, political, and legal environment influencing life opportunities and restoring the biographical stance for regaining control of persons’ lives, imprisonment is a political tool and a for-profit enterprise of special interest groups [2].

As the history of the influenza pandemics of the 20th Century—A/H_1_N_1_ in 1918 to 1919, A/H_2_N_2_ in 1957, A/H_3_N_2_ in 1958—and those of the 21st Century—SARS-CoV-1 in 2003, A/H_1_N_1pdm_ in 2019, and MERS in 2012—the SARS-CoV-2 pandemic is not over until transmission is interrupted in all settings. The synergistic combination of the high-transmissibility of the novel SARS-CoV-2 and the high flow into and out of jails will continue to threaten those imprisoned, the staff, and the larger community [14]. Facing the COVID-19 pandemic calls for worldwide efforts to include joint planning by public health institutions with federal, state, and local authorities to explicitly and transparently implement and monitor preventive and mitigation interventions in correctional facilities [14]. Depopulating jails and prisons during this pandemic is the only means of achieving meaningful social distancing and protecting medically vulnerable persons. To implement effective population management approaches such as sequestration, isolation, and quarantine practices, expanding testing of prisoners and correctional officers is critical inside these facilities.

In the long term, policy-makers need to address what this pandemic has uncovered and what we have chosen to neglect: the existence of underlying unfair social and economic structures that are tightly bound to unfair health outcomes in the US and elsewhere. The disproportionate impact of COVID-19 on US prisons and jails is part of a larger pattern of the health disparity aspects of this viral illness. For example, in US Southern states most of the COVID19 deaths now occur among African American populations [15]. Though, in the short term, and while the pandemic continues to reshape the daily lives of all citizens in every corner in the world, no one is safe until everyone is safe, including those who are currently incarcerated. An effective response to prevent and mitigate the COVID-19 impact in custodial settings is a pivotal component of the global response to this pandemic.
